# Supplementary material for: Bone histology and growth curve of the earliest ceratopsian Yinlong downsi from the Upper Jurassic of Junggar Basin, Northwest China
Source: PeerJ. 2024 Dec 19;12:e18761. doi: 10.7717/peerj.18761 (PMC11663408; doi:10.7717/peerj.18761)
Supplement: Supplemental Information 1 [file peerj-12-18761-s001.docx]

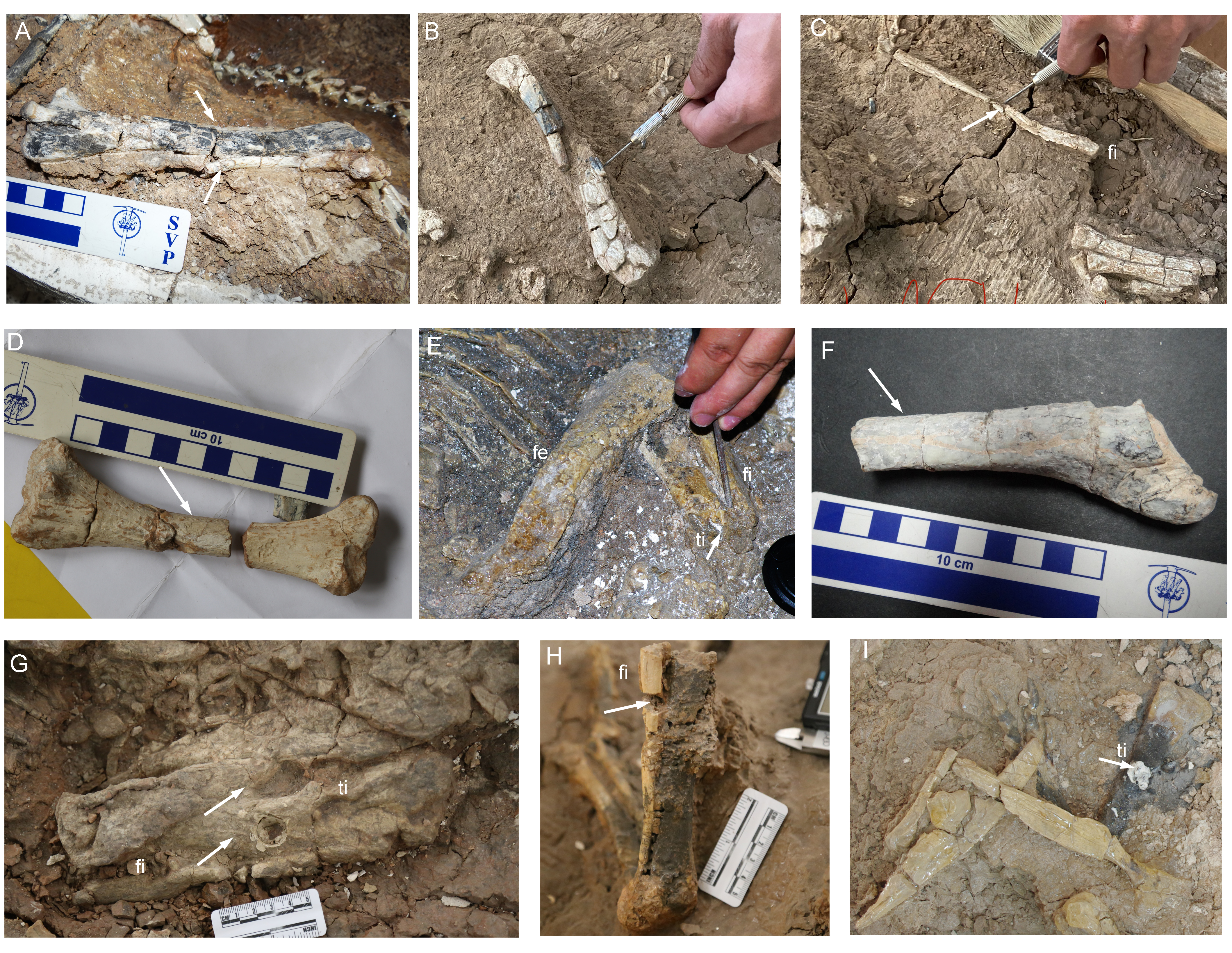


Fig. S1. Sactional position of *Yinlong downsi* . **A**. IVPP V18677, left tibia and fibula; **B-C**. IVPP V33266, B. Tibia in posterior view; C. fibula in medial view. **D**. IVPP V18678, left tibia in anterior view; **E**. IVPP V18636, showing articulated femur, proximal tibia, and fibula. **F**. IVPP V18679, left tibia in posterior view. G. IVPP V18683, articulated right tibia and fibula in anterior view. **H**. IVPP V18682, partial right tibia and fibula in posterior view. **I**. IVPP V18637. All the white arrows denote sectioned positions. **Abbreviations**: **fe**, femur; **fi**, fibula; **ti**, tibia


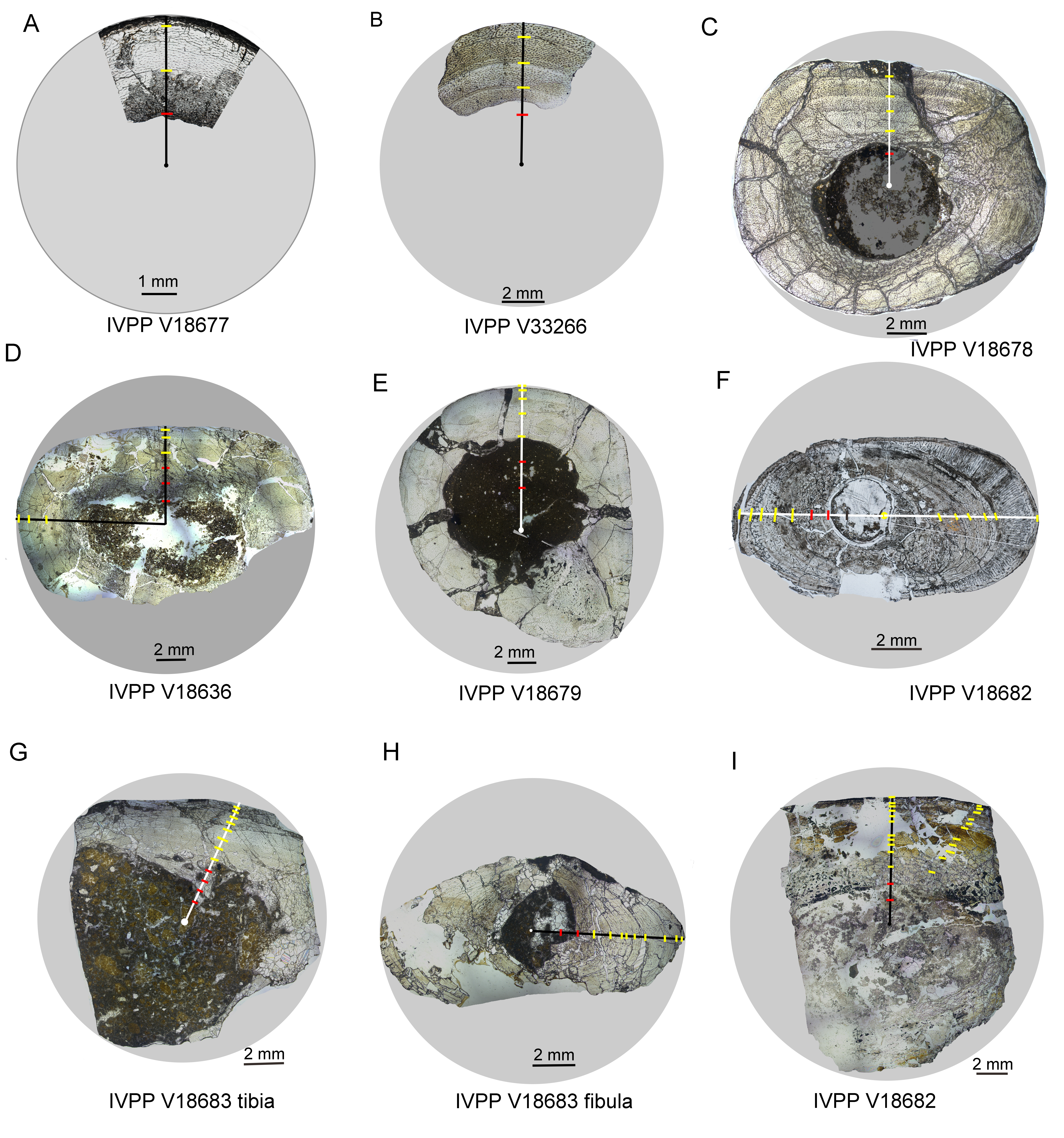


Fig. S2. Age estimation was calculated by using the extrapolation method. The cross sections of all the tibiae are about elliptical, and the midpoint of each bone is the center of the smallest circle that covers the oval. **A.** IVPP V18677; **B.** IVPP V33266; **C.** IVPP V18678, tibia; **D.** IVPP V18636, tibia; **E.** IVPP V18679, tibia; **F.** IVPP V18682, fibula; **G.** IVPP V18683, tibia; **H.** IVPP V18683 fibula; **I.** IVPP V18682, tibia. Yellow colors denote the actual LAGs, and the red colors denote the estimated LAGs.
